# Acute Effects of Whole Body Vibration on Inhibition in Healthy Children

**DOI:** 10.1371/journal.pone.0140665

**Published:** 2015-11-02

**Authors:** Anne E. den Heijer, Yvonne Groen, Anselm B. M. Fuermaier, Marieke J. G. van Heuvelen, Eddy A. van der Zee, Lara Tucha, Oliver Tucha

**Affiliations:** 1 Clinical and Developmental Neuropsychology, University of Groningen, Groningen, The Netherlands; 2 Center for Human Movement Sciences, University of Groningen, University Medical Center Groningen, Groningen, the Netherlands; 3 Center of Behaviour and Neuroscience, Department of Molecular Neurobiology, University of Groningen, Groningen, the Netherlands; University of Akron, UNITED STATES

## Abstract

**Objectives:**

Whole Body Vibration (WBV) is a passive exercise method known to have beneficial effects on various physical measures. Studies on adults furthermore demonstrated beneficial effects of WBV treatment on cognition (e.g. inhibition). The present study replicated these findings in healthy children and examined acute effects of WBV treatment on inhibition.

**Methods:**

Fifty-five healthy children (aged 8–13) participated in this within-subject design study. WBV treatment was applied by having the children sit on a chair mounted to a vibrating platform. After each condition (vibration vs. non-vibration), inhibition was measured by using the Stroop Color-Word Interference Test. Repeated measures analyses were applied in order to explore the effects of WBV treatment on inhibition, and correlations were computed between the treatment effect and participant characteristics in order to explore individual differences in treatment sensitivity.

**Results:**

Three-minute WBV treatments had significant beneficial effects on inhibition in this sample of healthy children. Especially the repeated application (three times) of WBV treatment appeared beneficial for cognition. Stronger WBV treatment effects were correlated with higher intelligence and younger age, but not with symptoms of Attention Deficit Hyperactivity Disorder (ADHD).

**Conclusions:**

This study demonstrates that especially repeated WBV treatment improves inhibition in healthy children. As this cognitive function is often impaired in children with developmental disorders (e.g. ADHD), future studies should further explore the effects, working mechanism and potential applicability of WBV treatment for this target group.

## Introduction

Whole Body Vibration (WBV) can be defined as a passive exercise training method that exposes the whole body to low frequency environmental vibration [1]. Performing dynamic exercises in combination with WBV is a well-known sports / fitness practice, but the here described passive application is realized by holding static poses during the vibration exposure [1]. The WBV is thus achieved by standing or sitting on an apparatus that creates the environmental vibration. This kind of application of WBV can be regarded as passive physical exercise because the body is moving (reflexive muscle contractions) without active performance. The known physical and physiological effects of WBV resemble those of active exercise, and include increased balance, mobility, posture control, oxygen uptake, heart rate, blood pressure, blood flow and muscle strength in healthy adults [2–6]. Beneficial effects of WBV have also been reported in adult samples with neurological conditions such as Parkinson’s Disease [7], Alzheimer’s Disease [8] and stroke [9]. Studies investigating the effectiveness of WBV for the improvement of physical fitness and the musculoskeletal system in children with physical/muscular disabilities (e.g. Duchenne muscular dystrophy) stressed that WBV is also a safe training method for children [10–12].

Studies on the effects of WBV on cognition, however, are scarce but the first results are promising. Two recent studies on samples of healthy adults showed that cognitive functioning (i.e. inhibitory control as a measure of attention) improved significantly when immediately assessed following two-minute WBV treatments [1,13]. Although preliminary, studies have also described improved cognition after WBV treatment in clinical samples. A study on patients with traumatic brain injury (TBI) and healthy adults showed beneficial effects of passive vibration of the forearm (proprioceptive stimulation) in both groups on a computerized neuropsychological assessment of attention (choice-reaction-time task), as well as neurophysiological measures (event-related potentials: ERP) [14]. Patients with TBI showed longer P300 latencies than healthy adults, that–specifically in the patient group–were shortened when receiving the vibration intervention. In addition, both speed and accuracy of target detection were enhanced. As this effect was seen in TBI patients but not in healthy individuals, these findings point to the possibility that WBV could be especially beneficial for cognitive processes of patients with neurological conditions. WBV effects are also found in adults with Attention Deficit Hyperactivity Disorder (ADHD), with two studies demonstrating acute as well as longer-lasting effects of WBV on cognition. First, Fuermaier and colleagues [1] showed that not only in healthy individuals but also in individuals with ADHD, inhibition improved when assessed shortly after receiving WBV treatments of two minutes. Second, Fuermaier and colleagues demonstrated longer-lasting effects (at least 16 hours) of WBV on several neuropsychological measures of attention and executive functions in a case report of an adult with ADHD [15]. Since WBV appears to specifically improve inhibition and attention which are both functions that are often impaired in patients with ADHD [16], WBV could be considered as a potentially new, effective and safe treatment option for ADHD. Because ADHD is a neurodevelopmental disorder that already presents in childhood [17], it is of utmost interest to not only explore the effectiveness of WBV treatment in adults but also in children. Considering the fact that ADHD is more prevalent in children than in adults [18–20], it is even more relevant to aim to elucidate the effects of WBV on cognition in children. WBV treatment might have the potential to constitute an additional or substitute intervention for pharmacological treatments (at least for some children). Pharmacological treatments are often associated with several undesirable effects including side-effects, incomplete response-rates and unclear (long-term) effects [21–30], emphasizing the constant search and current need for safe and effective treatment options for children with ADHD. In contrast, the literature on the physical effects of WBV treatment on different groups of children indicated the safety of WBV [10–12]. In case that WBV treatment indeed improves aspects of cognition in children, children with neurodevelopmental or neurological conditions other than ADHD might also benefit from WBV treatment.

The main aim of the present study is to examine whether WBV has acute effects on cognition, more specifically inhibition, on healthy 8–13 year-old children. This study replicates and extends previous studies describing positive effects of WBV on cognition in healthy adults [1,13,15]. Based on previous findings, it is expected that cognitive functioning improves in healthy children when assessed immediately following WBV treatment. A secondary objective is to explore possible effects of treatment repetition, as previous studies demonstrated prolonged effects of WBV treatment [15]. The expectation is that repeated WBV applications lead to an accumulated positive effect on cognition. The last objective is to explore whether the magnitude of the treatment effect depends on participant characteristics, such as the age of the child and the number of subclinical ADHD symptoms.

## Methods

### Participants

Fifty-five 8–13 year-old healthy children (27 males, 28 females) participated in the study, and had a mean age of ten years (see Table 1 for sample characteristics). The sample was recruited via a primary school (n = 28) and via a public announcement in a local newspaper (n = 27). Participation was voluntary and no monetary compensation was offered, but the children received a small gift after the assessment (e.g. crayons, stuffed animal). None of the parents/caregivers of the children reported a history of developmental, neurological or psychiatric disease in the child on parent-reported questionnaires. For one child, parents reported that it had been diagnosed with dyslexia, nevertheless the child was included since the teacher judged the child’s current reading level as sufficient to perform the experiment. At the time of the study, none of the children were taking medication known to affect the central nervous system. Furthermore, no participant suffered from a red-green color deficiency as assessed by a form of the Ishihara Color Test [31], which was a prerequisite to carry out the Stroop test in this study (see Materials). Prior to the assessment, parents completed an ADHD symptom questionnaire (*ADHD Vragenlijst*: AVL; see materials) [32] and the Child Behavior Checklist (CBCL; see materials) [33]. Based on the AVL, children can be screened for presenting a clinical level of ADHD symptoms (AVL total cut-off score > 42). CBCL scores indicate whether children display behavior problems in a clinical range on a total, internalizing and externalizing behavior scale. One child scored in the clinical range of the AVL as well as the CBCL. This child was nevertheless included since there was no formal diagnosis of ADHD. Furthermore, statistical exploration of the sample revealed no outlying characteristics of this child. Intelligence was estimated by means of a shortened version of the Wechsler Intelligence Scale for Children-III (WISC-III), using one perceptual reasoning subtest (Block Design) and one verbal comprehension subtest (Similarities) [34]. The mean group scores on all tests and questionnaires are presented in Table 1.

**Table 1:** Characteristics of the sample with healthy children (n = 55, 27 males).

	M±SD	Range (min-max)
Age (years)	10.1±1.2	8–13
TIQ ^a^ estimate (norm score)	108±19	65–145
VIQ ^b^ estimate (norm score)	106±17	64–145
PIQ ^c^ estimate (norm score)	107±24	55–144
AVL ^d^ total score (raw score)	10.5±11.2	0–65
CBCL ^e^ total score (t-value)	17.8±16.6	1–67
CBCL ^e^ internalizing score (t-value)	5.0±6.0	0–25
CBCL ^e^ externalizing score (t-value)	4.8±5.8	0–23

^a^ Total IQ as measured by the WISC-III

^b^ Verbal IQ as measured by the WISC-III Similarities subtest

^c^ Performance IQ as measured by the WISC-III Block-Design subtest

^d^ Parent-report ADHD questionnaire

^e^ Parent-report Child Behavior Checklist (2 missing values)

#### Ethics statement

This study was approved by the Ethical Committee Psychology (ECP) affiliated to the University of Groningen, The Netherlands. All parents signed an informed consent form prior to the assessment, as did children aged 12 or older. Parents were debriefed after the assessment by receiving a summary of the group findings and/or their child’s outcomes (as preferred). All parents and children were informed that participation was voluntary and that they had the right to withdraw, stop or pause participating in the study at all times.

### Materials

#### Whole Body Vibration

**Fig 1:**
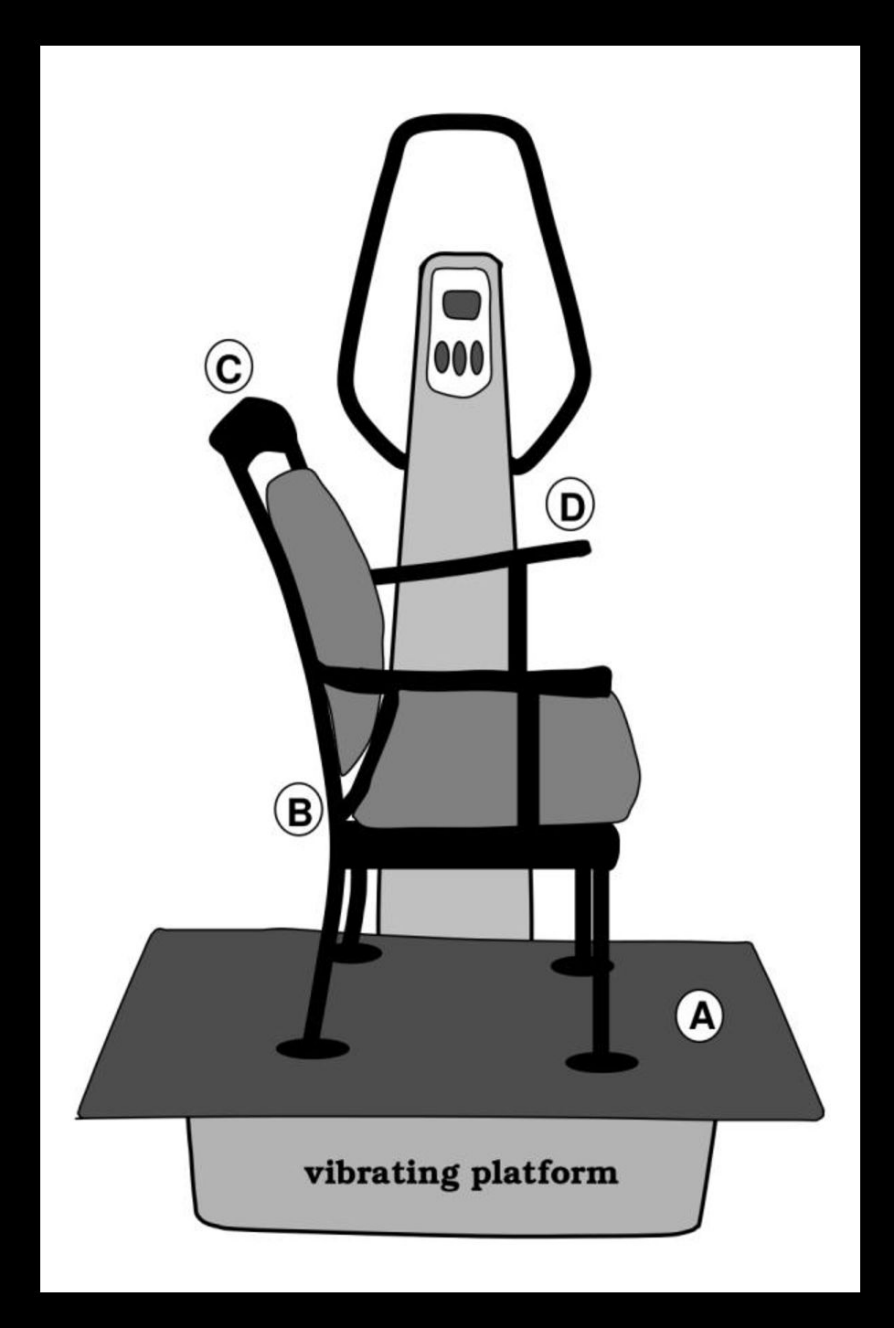
Image of the vibrating platform and chair. The vibrating platform with the attached wooden platform and mounted chair. The actual vibration frequency and amplitude were measured at locations A, B, C and D (Figure adopted with persmission from Fuermaier et al., 2014a).

Passive WBV was applied by having the children sit on a chair that was mounted on a vibrating platform (*Vibe 300*, Tonic Vibes, Nantes, France). In order to mount the chair to the *Vibe 300*, a wooden platform (0.5m x 0.9m) was attached to the *Vibe 300* first. To keep deviations from the vibrating frequency and amplitude as limited as possible, the wooden platform and the chair were attached to the *Vibe 300* with bolts from underneath. The *vibration frequency* was set at *30Hz* and the *vibration amplitude* was set at *4mm*, based on the manufacturer settings and pilot studies demonstrating beneficial effects of WBV using this amplitude and frequency [1,13,15]. This setup was considered comfortable for the participants. However, some deviations from the manufacturer setting of the vibration frequency and amplitude were assumed as a result of the mounted platform and chair on the *Vibe 300*. The actual vertical displacements (frequency and amplitude) were consequently measured on different locations of the chair (see A, B, C and D in Fig 1), based on acceleration data without a person on the *Vibe 300*. The measured displacements (frequency/amplitude) were 30Hz/0.44mm (location A), 30Hz/0.44mm (location B), 30Hz/0.6mm (location C) and 30Hz/0.50mm (location D) [1]. The modified *Vibe 300* was located in a quiet testing room at respectively a primary school in Grijpskerk, The Netherlands, and the Department of Clinical and Developmental Neuropsychology of the University of Groningen, The Netherlands.

#### Measurement of inhibition

The Stroop Color-Word Interference Test (Stroop, 1935) was applied to explore the effects of WBV on inhibition, which constitutes an important aspect of attention. We selected this neuropsychological test as it has been shown to be a valid measure [35] that is furthermore sensitive to the effects of WBV [1,13,15]. The present study made use of two of the three conditions of the Stroop Test: the Color-Block test and the Color-Word Test. In the Color-Block Test, a card was presented with twenty squares that were either yellow, blue, green or red. Children were asked to name the colors of the squares as fast as possible, from left to right and top to bottom. In the Color-Word Test, a card was presented with a list of 52 color names (yellow, blue, green, red), however, the ink color was different from the color names (e.g. the word yellow was printed in red). The participant’s task was to name the ink colors of the word as fast as possible, from left to right and top to bottom, thereby ignoring the written name of the color. The time to complete each test was measured by a stopwatch and errors or corrections were noted but not included in the analysis. The score on the Color-Block Test can be seen as a measure of psychomotor speed. The time difference between the Color-Word Test and the Color-Block Test can be regarded as a measure of inhibitory control, i.e. how well a person can inhibit the interference of the automatic response tendency of reading the word. Shorter completion times thus stem from less interference and represent better inhibition. In order to be able to apply the tests in a repeated measures design, 10 parallel versions (for four practice trials and six experiment trials) were used.

#### Parent-reported measures of ADHD symptoms and behavior

The parent(s)/caretaker(s) of the participating children were asked to fill out a Dutch ADHD symptom questionnaire about their child (*ADHD Vragenlijst*: AVL) [32]. The AVL is based on the diagnostic criteria for ADHD of the Diagnostic and Statistical Manual of Mental Disorders (DSM-IV) and consists of 18 questions covering the three ADHD dimensions: inattention, hyperactivity and impulsivity [36]. On a five-point scale, the parents indicated the frequency of the child’s behavior in the past year (0 = never; 1 = sometimes; 2 = regularly; 3 = often; 4 = very often).

Furthermore, the CBCL was applied to identify a child’s potential (problem) behavior [33]. The questionnaire measures emotional, behavior and social domains with 100 items that are scored on a three-point scale (0 = not true; 1 = somewhat/sometimes true; 2 = very/often true). The total score on the CBCL can be subdivided into (I) an internalizing problems total score, (II) an externalizing problems total score and (III) a total problems score.

### Procedure

Prior to the start of the assessment, the children were introduced to the test situation and the *Vibe 300*, in order to make them feel at ease. First, children were asked to perform the Ishihara Color Test [31] to check for red-green color deficiencies. Second, two subtests of the WISC-III were performed (Block Design and Similarities). After these basic assessments, the experiment started with four practice trials of the Stroop Color-Word Interference Test (without WBV) in order to minimize practice effects during the experimental trials. Four parallel versions of the test were used for these practice trials, separated by a resting period of three minutes between each trial. The children were then asked to take place on the chair on the *Vibe 300*, and instructed to sit in upright position throughout the whole experiment, with the arms and hands on the rest and both feet on the platform, and to keep body movements to a minimum [1]. The experiment itself consisted of six trials. Each trial started with a three-minute period which was either (A) a period of WBV (vibration condition), or (B) a resting period of no vibration (non-vibration condition). Immediately after the experimental treatment, first the Color-Word Test and then the Color-Block Test was applied. Each trial finished with a break of three minutes. During the non-vibration conditions the level of physical and cognitive activity was kept to a minimum by instructing the child to sit quietly on the chair. The order of the experimental treatments was as follows: A-B-B-A-B-A. The six experimental trials thus comprised three trials of WBV (trials 1, 4 and 6) and three resting periods (trials 2, 3 and 5) that were separated by breaks of three minutes. This sequence of the trials was chosen in order to balance rank order effects / practice effects when analyzing the two treatment conditions. The total duration of the assessment was circa ninety minutes per individual.

### Statistical analysis

Mean duration of completion of the Color-Word Test and Color-Block Test was computed for the three trials with WBV and for the three resting trials. A repeated measures ANOVA was performed with the factors ‘WBV treatment’ (vibration, non-vibration) and ‘Stroop condition’ (Color-Word Test, Color-Block Test). Significant interaction effects were specified by conducting paired t-tests with the factor WBV treatment for the Color-Word Test and the Color-Block test separately. Furthermore, in order to explore potential repetition effects of WBV treatment, an additional analysis compared inhibition effects *over the course* of the trials. For this purpose, a repeated measures ANOVA was conducted with ‘repetition’ as an additional factor, with the levels ‘first’ (trial pair 1 and 2), ‘second’ (trial pair 3 and 4), and ‘third’ (trial pair 5 and 6). Finally, the association between the WBV treatment effect and characteristics of the child was explored by applying a multiple linear regression analysis using the ‘forward’ method with age, gender, total IQ (TIQ) and total AVL-score as predictors and a WBV treatment effect measure as the outcome variable. The WBV treatment effect measure was computed by subtracting the inhibition effect (Color-Word minus Color-Block time) in the vibration condition from the non-vibration condition, with positive scores consequently indicating enhanced inhibition in the vibration condition compared to the non-vibration condition and negative scores indicating reduced inhibition in the vibration condition compared to the non-vibration condition. To further examine the effects, effect sizes were firstly calculated by using Cohen’s d and its interpretation classification index [37], describing small effects (0.2 < d < 0.5), medium effects (0.5 < d < 0.8), and large effects (d > 0.8). Secondly, as also described by Cohen [37], η^2^ is a function of the effect size index f. A small effect size (f = .10) corresponds to an η^2^ = .01, a medium effect size (f = .25) to an η^2^ = .06 and a large effect size (f = .40) to an η^2^ = .14. A significance level of α = .05 was applied for all analyses. We checked whether outliers were present in the outcome measures, and only one child obtained an effect that exceeded a value of two standard deviations above the mean. Excluding this child did not change the results and therefore it was kept in the analysis. Boxplots of the difference scores for each measurement moment display the distribution of the scores and also show that there are no (non-linear) rank order effects that might have distorted our analyses and interpretations (see S1 Fig).

## Results

### Effects of WBV treatment on inhibition

**Fig 2:**
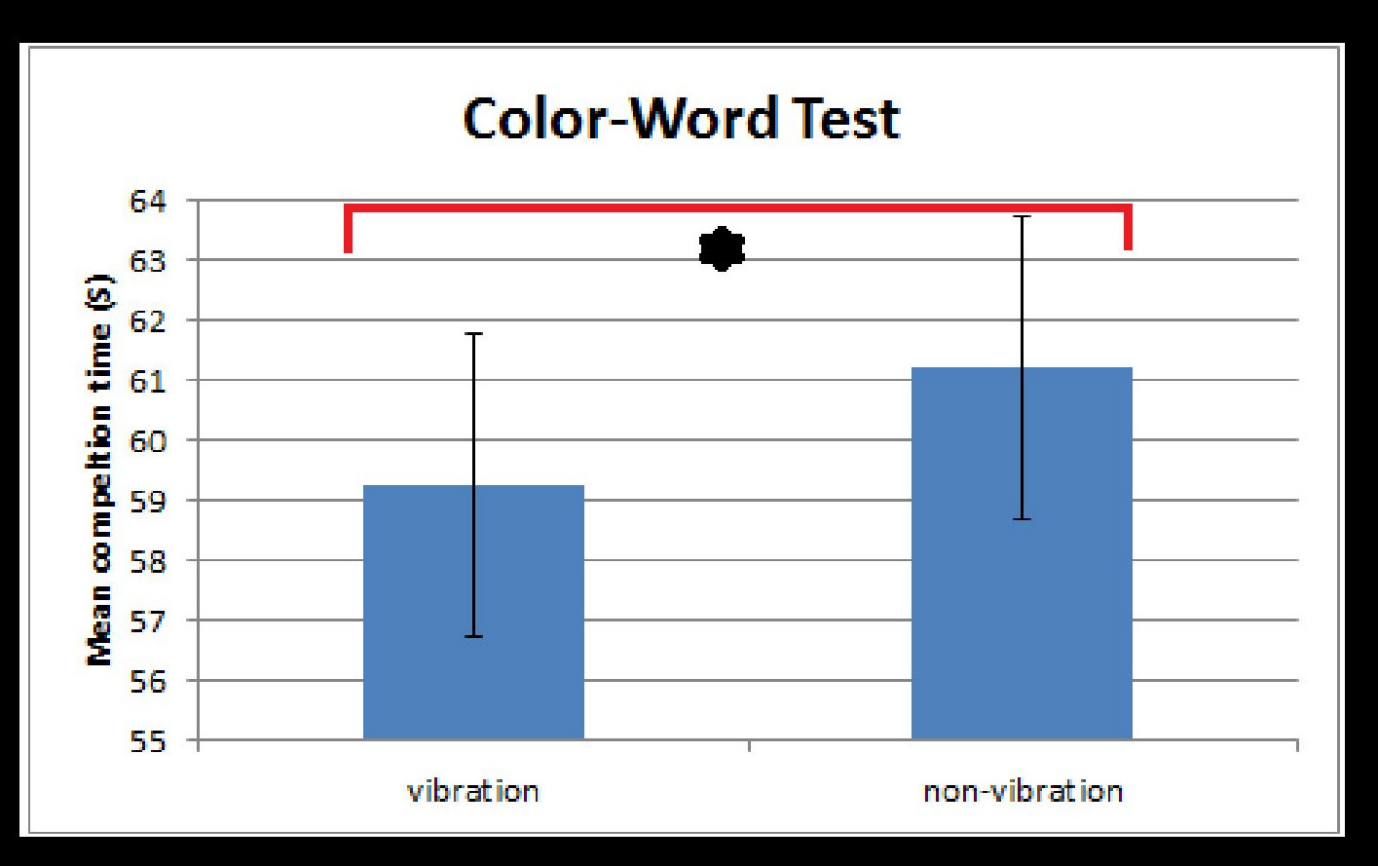
Stroop Color-Word times for vibration and non-vibration conditions. Mean completion times on the Stroop Color-Word Interference Test separated for the vibration and non-vibration conditions. Lower mean completion times represent less interference and a better inhibition. * = significant effect at p = .009

As expected, the children had longer completion times of the Color-Word Test than the Color-Block Test, as was demonstrated by a large main effect of Stroop condition, F(1, 54) = 408.03, p ≤ .001, η_p_^2^ = .88. This is indicative of a strong interference effect of naming the word meaning over naming the color of the ink. A significant interaction effect of ‘WBV treatment’ with ‘Stroop condition’ (F(1.00, 54.00) = 7.90, p = .007, η_p_^2^ = .13) indicated that the interference effect differed between the vibration and non-vibration condition with a medium effect size. Paired t-tests per Stroop condition showed a significant reduction in the completion time (seconds) of the Color-Word Test in the vibration condition (M = 59.25, SD = 17.82) compared to the non-vibration condition (M = 61.19, SD = 20.34), t(54) = -2.73, p = .009) (see Fig 2). Effect size calculations revealed that this effect was small-negligible (d = 0.10). Performances in the Color-Block Test did not differ significantly between the vibration condition (M = 44.74, SD = 15.28) and the non-vibration condition (M = 43.92, SD = 15.28), t(54) = 0.78, p = .44 (d = -0.05). The results did not change when the one child with dyslexia was excluded. These findings demonstrate a specific improvement of inhibitory control by WBV treatment.

### Exploration of repetition effects of WBV treatment

**Fig 3:**
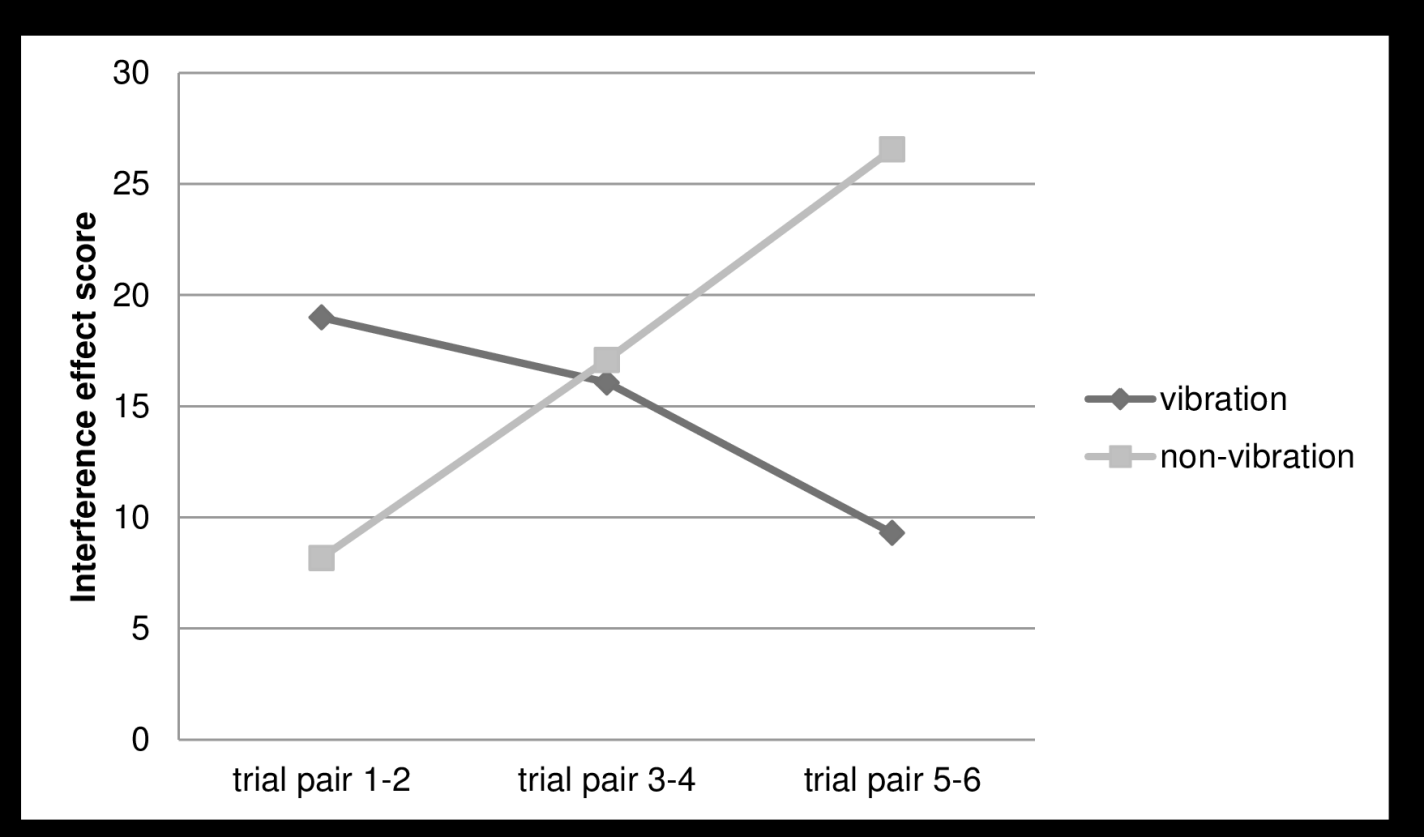
Interference effects for treatment repetition. Interference effects in the Stroop Color-Word Interference Test separated for the vibration and non-vibration conditions and repetition of the treatment. Lower mean completion times represent less interference and a better inhibition.

As can be seen in Table 2, an exploratory analysis of repetition effects of the treatment conditions revealed that children showed significantly longer completion times on the Stroop Test with test progression, indicating a general decrease of performance. As indicated by a significant ‘repetition’*’Stroop condition’ interaction, this performance decrement appeared to be specific for the Color-Word Test. While mean completion times increased with repetition during the execution of the Color-Word Test (first: M = 58.10, SD = 15.81; second: M = 60.14, SD = 19.50; third: M = 62.42, SD = 22.86), the mean completion times remained stable for the Color-Block Test (first: M = 44.52, SD = 15.22; second: M = 43.56, SD = 14.94; third: M = 44.49, SD = 15.89). This means that specifically inhibition decreases with test progression, and not performance speed. Interestingly, the performance decrease in the Color-Word Test was dependent on the ‘WBV’ condition (WBV treatment), as indicated by the strongly significant ‘repetition’*’Stroop condition’*’WBV treatment’ interaction which represented a large effect size. Exploring this three-way interaction showed a decrease of the interference effect (i.e. improvement of inhibitory control) in the vibration condition and an increase of the interference effect (i.e. reduction of inhibitory control) in the non-vibration condition with test progression. This is demonstrated in Fig 3: the interference effect score (I) is significantly higher in the vibration condition (M = 18.99, SD = 7.20) than in the non-vibration condition (M = 8.18, SD = 5.41) in Trial pair 1 and 2, t(54) = 9.90, p ≤ .001; (II) does not differ significantly across the vibration condition (M = 16.07, SD = 11.25) and the non-vibration condition (M = 17.09, SD = 11.09) in Trial pair 3 and 4, t(54) = 0.53, p = .595; and (III) is significantly lower in the vibration condition (M = 9.30, SD = 8.67) than in the non-vibration condition (M = 26.55, SD = 16.26) in Trial pair 5 and 6, t(54) = 8.14, p ≤ .001. Inhibition thus improved after three vibration trials whereas it diminished after three non-vibration trials. Effect size calculations revealed a reverse effect of WBV treatment on interference scores in Trial pair 1 and 2 (d = -1.70), a negligible effect in Trial pair 3 and 4 (d = 0.14) and a large effect in Trial pair 5 and 6 (d = 1.32).

**Table 2:** Outcomes of the repeated measure analysis of repetition effects.

Repetition	F(2,108) = 5.4, p = .008, η_p_^2^ = .09
Repetition x Vibration condition	F(2,92) = 30.9, p ≤ .001, η_p_^2^ = .36
Repetition x Interference condition	F(2,93) = 5.3, p = .009, η_p_^2^ = .09
Repetition x Vibration condition x Interference condition	F(2,92) = 56.9, p ≤ .001, η_p_^2^ = .51

### Relations between WBV treatment effect and demographic variables

Based on the regression analysis of the participant characteristics age, gender, TIQ and AVL-score, no significant predictors were found for the WBV treatment effect on inhibition. Further exploration of correlations of these variables with the WBV treatment effect revealed only a significant positive correlation with TIQ (r = .24, p = .04). This indicates that the improvement of inhibition by WBV treatment was somewhat stronger in children with higher total IQ scores.

## Discussion

The present study demonstrated that WBV treatment improves inhibition in healthy 8–13 year-old children. Three repeated treatments with WBV of three minutes were found to benefit inhibition (i.e. inhibitory control as measured by the Stroop Color-Word Test), showing acute effects of WBV compared to the non-vibration condition. The effects were specifically effective for controlled cognitive functions, with improved inhibition of automatic response tendencies, but unchanged automatic cognitive functions (i.e. speed of color naming). This study thus replicated findings of improved cognition such as inhibition and attention after WBV treatment in healthy adult samples [1,13] in a sample of healthy children. Replication in children demonstrates that the efficacy of WBV is probably not attributable to a placebo effect. In studies on the effect of WBV treatment on cognition in adults, potential expectancy effects are a substantial limitation since hypothesized effects of WBV treatment are easily understandable for adults (also for non-informed participants) and they could therefore behave accordingly. This appears unlikely in children. The present study therefore not only shows beneficial effects of WBV treatment on cognition in children but also amplifies general findings that WBV can benefit cognition. WBV studies in adults however should use proper placebo conditions. The fact that already a low number of short periods of WBV treatments is beneficial for inhibitory control is promising and encourages future studies to further examine both short- and long-term effects of WBV on cognition. The finding that WBV treatment can improve controlled cognitive processes in a sample of *healthy* children (aged 8–13 years) shows that WBV is a promising new treatment that could even be applied as a cognitive enhancement treatment in children with normal baseline cognitive functioning. Such cognitive enhancements could be relevant when optimal cognitive functioning (e.g. with regard to distractibility) is desired, for instance during school tests, but it might also generally benefit cognitive development. This is of particular relevance since better cognitive functioning is generally associated with higher academic achievement, socioeconomic status and social integration, and can serve as a buffer against cognitive impairment [38,39].

Several hypotheses are currently discussed regarding the working mechanism of WBV treatment. One hypothesis regards sensory stimulation, stating that sensory stimulation of WBV may affect neurotransmission in especially sensory brain regions, the prefrontal cortex and the hippocampus, thereby benefiting cognition [13]. Another hypothesis is that WBV changes muscle length which in turn would stimulate muscle spindles, reflex responses and muscle activity, increasing oxygen uptake and heart rate [3]. Studies on the effect of *active* physical exercise suggested that the underlying working mechanism entails enhanced neural growth and development [40–44], increased levels of catecholamines and proteins/enzymes (e.g. dopamine, tyrosine hydroxylase and Brain-Derived Neurotrophic Factor) [45–48], and positive influences on the dorsolateral PFC [45]. Whether this holds true for *passive* exercise (WBV) as well has to be clarified in future studies. Several animal studies explored the underlying mechanism of improvements in cognition (e.g. spatial memory) following repeated application of WBV in mice [49–52]. The results of these studies suggest that WBV activates/increases the following mechanisms: (I) the activity of the cholinergic system in the forebrain, (II) glucose-transportation across the blood-brain barrier, (III) the expression of immediate early genes (enhancing neuron responsiveness), (IV) production of proteins required for neuronal plasticity, and (V) neurogenesis (for a summary see [1]). Furthermore, it was suggested that (VI) WBV increases the enzyme tyrosine hydroxylase which helps catalyze the synthesis of a precursor of dopamine which affects movement, motivation and cognition [53–55]. Lastly, it is conceivable that WBV, like active physical exercise, increases overall arousal which is in turn related to immediate cognition-enhancing effects. This would imply dissipating gains in cognitive functioning with longer spacing between WBV treatment and testing. Future studies should further examine this arousal component of WBV and link it to effect durations.

The present findings showed that especially the *repeated application* of WBV treatment is beneficial for cognition, with an increase in inhibitory control following repeated WBV treatment, and a decrease in inhibitory control resulting from repeated assessment without WBV. Surprisingly, a reversed effect was seen in the first trial, with better inhibitory control in the non-vibration condition than following WBV treatment. Children were possibly initially distracted by the vibrations and gradually got used to it. Regarding the increased inhibitory control after repeated WBV treatment, it is conceivable that accumulation of WBV is necessary for a beneficial effect. We speculate that the above-mentioned neurophysiological effects need time (at least more than two occasions of three minutes of WBV treatment) to unfold their effects, but future studies should examine ideal treatment periods. A case-study on an adult with ADHD demonstrated prolonged effects on cognitive performance (at least 16 hours) after repetitive WBV applications [15]. However, these effects were not seen at a follow-up assessment that was performed two weeks after discontinuation of WBV treatment. As we found the strongest effect in the last WBV trial, it should be examined whether *longer* and/or *repeated* WBV application results in more beneficial effects and how long these effects last. Potential distressing effects of the WBV treatment on the children in the present study cannot be ruled out, as inhibition scores were lower in the vibration condition than in the non-vibration condition during the first trial, comparable in the second trial and higher in the third and last trial. This observation points to a possible habituation effect to, for instance, the (physical) experience and noise of the WBV application. Future studies should aim to prevent habituation effects by getting participants accustomed to the experience of WBV before starting the experiment.

Regarding participant characteristics, it was found that the WBV treatment effect was positively related to IQ, suggesting potential stronger WBV treatment effects in children with higher intelligence. This may be explained by the relation between higher intelligence and better developed levels of executive functions, allowing more possibilities for improvement in this domain [56]. No significant correlations between WBV treatment effects and age, gender or parent-reported ADHD scores were found. The absence of a correlation between WBV treatment and ADHD symptom scores could be attributable to a low level of variance, with most of the children presenting low levels of ADHD symptoms. The improvements of controlled cognitive processes following WBV treatment found in the present study are nevertheless considered relevant for clinical groups presenting attention and inhibition deficits, because an improved cognitive functioning induced by WBV treatment has already been demonstrated in previous research on adults with TBI [14] and in adults with ADHD, who showed larger beneficial effects than healthy individuals [1,15]. The positive effects of WBV treatment on inhibitory control as found in the present study is of particular relevance for children with ADHD, since they often present with problems in inhibition (e.g. see[16,57]). Because safe, cost-effective and easy-to-apply treatment methods for children with ADHD are sought-after, the cognitive as well as behavioral effects of WBV should be further investigated. Current data indicate that WBV treatment might represent a safe, easy to apply, inexpensive and effective treatment. In children with ADHD, for example, WBV treatment could be applied in addition to or instead of pharmacological treatments that can have several disadvantages and uncertainties including long-term effects, side-effects and incomplete response rates [21–30]. WBV treatment might also be favorable to physical exercise to some children, as the latter may have similar effects but appears less flexible, more time-consuming and restricted to those who are fit enough to perform physical exercise [13].

### Limitations and future directions

The present study should be viewed in the light of some limitations. The first limitation concerns the limited measurement of cognitive functioning, as the only assessed measure in the present study was inhibition. The possible generalizability to broader cognitive or behavioral improvement therefore remains uncertain. However, a case-study in an adult with ADHD showed long-term benefits of WBV treatment on multiple cognitive functions including vigilance, flexibility, working memory and inhibition [15]. This strengthens our recommendation to apply more neuropsychological, behavioral and physical measures in future studies in order to examine which functions are to what extent affected by WBV. A second limitation regards the fact that optimal application conditions remain unknown, as well as potential clinical groups that could benefit from such a treatment. Determining reasonable application schedules (i.e. regarding time, frequency and duration of WBV treatment) and its effects (i.e. short versus long-term) would support the development of WBV treatments for clinical groups. It would thus be interesting to explore for instance (I) the effects of vibration of specific body parts (e.g. the arms and hands) on cognition, (II) the effectiveness of different durations, amplitudes and frequencies of WBV, (III) the influence of noise of the vibrating device (adding a no-vibration but comparably noisy condition), (IV) the effects of WBV treatment on cognitive functioning of clinical groups known to suffer from higher order cognitive problems such as a lack of attention and inhibition and (V) acute versus chronic effects of WBV treatment on cognition. The present study showed significant effects following WBV treatment in healthy children with potentially a ceiling effect regarding the improvement in inhibition. This could imply that cognitive gains in clinical groups of children might even be larger or, at least, normalize cognitive performance (as shown in an adult ADHD sample) [1]. Another limitation concerns the imbalanced treatment order applied in this study. Our choice to implement an A-B-B-A-B-A presentation order was in hindsight suboptimal compared to a fully symmetrical design in which half the participants would be tested in the opposite order of B-A-A-B-A-B. Future studies should apply symmetrical presentation orders, to avoid concerns of rank order effects. A final limitation might concern the lack of physical (e.g. weight, physical fitness) and psychological (e.g. sensory sensitivity) measures included for the assessment of moderating effects of WBV treatment on cognition. Sensory sensitivity is defined as an inherent characteristic related to individual differences in the detection of and reaction to sensory information (e.g. sound, touch, vision) [58]. As WBV may affect neurotransmission in sensory brain regions, the prefrontal cortex and the hippocampus through sensory stimulation [13], future studies should consider the participants’ sensory sensitivity in relation to the WBV treatment effect.

In conclusion, this first study on the effects of WBV treatment on cognition in healthy children demonstrated promising effects on inhibition. Interestingly, it was found that especially the *repeated application* of WBV treatment appears beneficial for inhibition, suggesting that the accumulation or longer duration of WBV is necessary for a beneficial effect. Exploratory correlational analyses suggested that more positive WBV treatment effects were seen in children with higher intelligence. Although ADHD-symptoms of the children were not significantly related to WBV effects (likely due to low variance of this measure), the improved inhibition function due to WBV is specifically relevant for children with ADHD, as they often present with problems in inhibition. Identifying the primary mechanisms of the cognitive enhancement, the cognitive benefits and reasonable application methods of WBV interventions, could help develop a new safe and effective treatment method for patients with cognitive problems, such as patients with ADHD and TBI.

## Supporting Information

S1 Fig Boxplots of the difference scores (= ‘diff’) for each measurement moment. Distribution of the scores.
(TIF)

## References

[1] Fuermaier A.B.M., Tucha L., Koerts J., Van Heuvelen M.J.G., Van der Zee E.A., Lange K.W., et al (2014a). Good vibrations–Effects of Whole Body Vibration on attention in healthy individuals and individuals with Attention–Deficit / Hyperactivity Disorder. PLoS ONE, 9(2), e90747 10.1371/journal.pone.0090747 24587412 PMC3938804

[2] Bogaerts A., Verschueren S., Delecluse C., Claessens A.L., Boonen S. (2007). Effects of whole body vibration training on postural control in older individuals: A 1 year randomized controlled trial. Gait Posture, 26, 309–316. 17074485 10.1016/j.gaitpost.2006.09.078

[3] Cardinale M., Bosco C. (2003). The use of vibration as an exercise intervention. Exercise and Sport Sciences Reviews, 31, 621–624. 10.1097/00003677-200301000-00002 12562163

[4] Cochrane D.J, Sartor F., Winwood K., Stannard S.R., Narici M.V., Rittweger J. (2008). A comparison of the physiologic effects of acute whole-body vibration exercise in young and older people. Archives of Physical Medicine and Rehabilitation, 89, 815–821. 10.1016/j.apmr.2007.09.055 18452726

[5] Lam F.M.H., Lau R.W.K., Chung R.C.K., Pang M.Y.C. (2012). The effect of whole body vibration on balance, mobility and falls in older adults: A systematic review and meta-analysis. Maturitas, 72, 206–213. 10.1016/j.maturitas.2012.04.009 22609157

[6] Stewart J.M., Karman C., Montgomery L.D., McLeod K.J. (2005). Plantar vibration improves leg fluid flow in perimenopausal women. American Journal of Physiology—Regulatory, Integrative and Comparative Physiology, 288, 623–629. 10.1152/ajpregu.00513.2004 15472009

[7] Pinto N.S., Monteiro M.B., Froes Meyer, P., Santos-Filho S.D., Azevedo-Santos F., Bernardo R.M., et al (2010). The effects of Whole Body Vibration exercises in Parkinson’s Disease: a short review. Journal of Medicine and Medical Science, 2 (1), 594–600.

[8] Da Cunha Sa-Caputo D., Da Costa P.R., Pacheco-Lima R., Kutter C., Costa-Cavalcanti R., Mantilla-Giehl P., et al (2014). Is Whole Body Vibration a viable option for individuals with Alzheimer’s Disease? Public Health Research, 4 (4), 136–143.

[9] Santos-Filho S.D., Monteiro M.O.B., Paiva D.N., Arnobio A., De Paoli S., Da Cunha Sa-Caputo D., et al (2014). Possible benefits of the Whole Body Vibration in the treatment of complications in stroke patients. British Journal of Medicine and Medical Research, 4 (7), 1539–1551.

[10] Matute-Llorente A., Gonzalez-Aguero A., Gomez-Cabello A., Vicente-Rodriguez G., Casajus Mallen, J.A. (2014). Effect of whole-body vibration therapy on health-related physical fitness in children and adolescents with disabilities: a systematic review. Journal of Adolescent Health, 54(4), 385–396. 10.1016/j.jadohealth.2013.11.001 24388109

[11] Myers K.A., Ramage B., Khan A., Mah J.K. (2014). Vibration therapy tolerated in children with Duchenne muscular dystrophy: a pilot study. Pediatric Neurology, 51(1), 126–129. 10.1016/j.pediatrneurol.2014.03.005 24830767

[12] Soederpalm A.C., Kroksmark A.K., Magnusson P., Karlsson J., Tulinius M., Swolin-Eide D. (2013). Whole body vibration therapy in patients with Duchenne muscular dystrophy–a prospective observational study. Journal of Musculoskeletal and Neuronal Interactions, 13(1), 13–18. 23445910

[13] Regterschot G.R., Van Heuvelen M.J., Zeinstra E.B., Fuermaier A.B.M., Tucha L., Koerts J., et al (2014). Whole body vibration improves cognition in healthy young adults. PLoS One, 9(6) 10.1371/journal.pone.0100506 PMC4065066 24949870

[14] Mueller S.V., von Schweder A.J., Frank B., Dengler R., Munte T.F., Johannes S. (2002) The effects of proprioceptive stimulation on cognitive processes in patients after traumatic brain injury. Archives of Physical Medicine and Rehabilitation, 83, 115–121. 11782841 10.1053/apmr.2002.27472

[15] Fuermaier A.B.M., Tucha L., Koerts J., van den Bos M., Regterschot G.R.H., Zeinstra E.B., et al (2014b). Whole-body vibration improves cognitive functions of an adult with ADHD: A case report. ADHD Attention—Deficit and Hyperactivity Disorders, 10.1007/s12402-014-0149-7 25031090

[16] Barkley R. A. (1997). Behavioral inhibition, sustained attention, and executive functions: Constructing a unifying theory of ADHD. Psychological Bulletin, 121(1), 65–94. 9000892 10.1037/0033-2909.121.1.65

[17] American Psychiatric Association. (2013). Diagnostic and Statistical Manual of Mental Disorders (5th ed.). Arlington, VA: American Psychiatric Publishing.

[18] Geissler J., Lesch K.P. (2011). A lifetime of Attention–Deficit / Hyperactivity Disorder: diagnostic challenges, treatment and neurobiological mechanisms. Expert Review of Neurotherapeutics, 11 (10), 1467–1484, 10.1586/ern.11.136 21955202

[19] Huang C.L., Chu C.C., Cheng T.J., Weng S.F. (2014). Epidemiology of treated Attention–Deficit / Hyperactivity Disorder (ADHD) across the lifespan in Taiwan: a nationwide population-based longitudinal study. PLoS One, 9 (4), e95014 10.1371/journal.pone.0095014 24736469 PMC3988191

[20] Willcutt E.G. (2012). The prevalence of DSM-IV Attention–Deficit / Hyperactivity Disorder: a meta-analytic review. Neurotherapeutics, 9 (3), 490–499. 10.1007/s13311-012-0135-8 22976615 PMC3441936

[21] Banaschewski T., Coghill D., Santosh P., Zuddas A., Asherson P., Buitelaar J., et al (2006). Long-acting medications for the hyperkinetic disorders. A systematic review and european treatment guideline. European Child and Adolescent Psychiatry, 15, 476–495. 16680409 10.1007/s00787-006-0549-0

[22] Biederman J., Newcorn J., Sprich S. (1991). Comorbidity of Attention–Deficit / Hyperactivity Disorder with conduct, depressive, anxiety and other disorders. American Journal of Psychiatry, 148(6), 564–577. 2018156 10.1176/ajp.148.5.564

[23] Buitelaar J., Medori R. (2010). Treating Attention–Deficit / Hyperactivity Disorder beyond symptom control alone in children and adolescents: A review of the potential benefits of long-acting stimulants. European Child and Adolescent Psychiatry, 19, 325–340. 10.1007/s00787-009-0056-1 19823900 PMC2843838

[24] Dela Pena I., Cheong J.H. (2013). Abuse and dependence liablity analysis of methylphenidate in the spontaneously hypertensive rat model of Attention–Deficit / Hyperactivity Disorder (ADHD): What have we learned? Archives of Pharmacal Research, 36(4), 400–410. 10.1007/s12272-013-0037-2 23471559

[25] Owens J.A., Maxim R., Nobile C., McGuinn M., Msall M. (2000). Parental and self-report of sleep in children with Attention–Deficit / Hyperactivity Disorder. Archives of Pediatrics & Adolescent Medicine, 154, 549–555. 10.1001/archpedi.154.6.549 10850500

[26] Sonuga-Barke E.J.S., Coghill D., Wigal T., DeBacker M., Swanson J. (2009). Adverse reactions to methylphenidate treatment for Attention–Deficit / Hyperactivity Disorder: Structure and associations with clinical characteristics and symptom control. Journal of Child and Adolescent Psychopharmacology, 19(6), 683–690. 10.1089/cap.2009.0024 20035586

[27] Stein M.A., Sarampote C.S., Waldman I.D., Robb A.S., Conlon C., Pearl, et al (2003). A dose-response study of OROS methylphenidate in children with Attention–Deficit / Hyperactivity Disorder. Pediatrics, 112(5), 404–413. 10.1542/peds.112.5.e404 14595084

[28] Taylor E., Dopfner M., Sergeant J., Asherson P., Banaschewski T., Buitelaar J., et al (2004). European clinical guidelines for hyperkinetic disorder–first upgrade. European Child and Adolescent Psychiatry, 13(1), 17–30. 10.1007/s00787-004-1002-x 15322953

[29] Van der Heijden K.B., Smits M.G., Gunning W.B. (2006). Sleep hygiene and actigraphically evaluated sleep characteristics in children with ADHD and chronic sleep onset insomnia. Journal of Sleep Research, 15, 55–62. 16490003 10.1111/j.1365-2869.2006.00491.x

[30] Wilens T.E., Adler L.A., Adams J., Sgambati S., Rotrose J., Sawtelle, et al (2008). Misuse and diversion of stimulants prescribed for ADHD: A systematic review of the literature. Journal of the American Academy of Child and Adolescent Psychiatry, 47(1), 21–31. 10.1097/chi.0b013e31815a56f1 18174822

[31] Ishihara S. (1917). Tests for color-blindness Tokyo: Hongo Harukicho.

[32] Scholte E.M. & Van der Ploeg J.D. (1998). Handleiding van de Vragenlijst ADHDproblemen. Lisse: Swets & Zeitlinger Testpublishers.

[33] Achenbach T.M., & Rescorla L. A. (2001). Manual for the ASEBA School-Age Forms and Profiles. Burlington, VT: University of Vermont, Research Center for Children, Youth, and Families.

[34] Wechsler D. (1991). The Wechsler intelligence scale for children—third edition. San Antonio, TX: The Psychological Corporation.

[35] Boonstra A.M., Oosterlaan J., Sergeant J.A., Buitelaar J.K. (2005). Executive functioning in adult ADHD: A meta-analytic review. Psychological Medicine, 35, 1097–1108. 16116936 10.1017/s003329170500499x

[36] American Psychiatric Association (APA). (1994). Diagnostic and statistical manual of mental disorders (4th edn). Washington DC: Author.

[37] Cohen J. (1988). Statistical power analysis for the behavioral sciences (2nd edn). Hillsdale: Erlbaum.

[38] Heckman J.J. (2011). The economics of inequality: The value of early childhood education. American Educator, 35, 31–35.

[39] Stern Y. (2009). Cognitive reserve. Neuropsychologia, 47, 2015–2028. 10.1016/j.neuropsychologia.2009.03.004 19467352 PMC2739591

[40] Best J. R. (2010). Effects of physical activity on children's executive function: Contributions of experimental research on aerobic exercise. Developmental Review, 30, 331–351. 21818169 10.1016/j.dr.2010.08.001 PMC3147174

[41] Bishop D. V. M. (2007). Curing dyslexia and Attention–Deficit / Hyperactivity Disorder by training motor co-ordination: Miracle or myth? Journal of Pediatrics and Child Health, 43(653), 655. 10.1111/j.1440-1754.2007.01225.x PMC2835859 17854448

[42] Bolduc V., Thorin-Trescases N., Thorin E. (2013). Endothelium-dependent control of cerebrovascular functions through age: exercise for healthy cerebrovascular ageing. American Journal of Physiology, 305(5), 620–633. 10.1152/ajpheart.00624.2012 23792680

[43] Halperin J.M., Bédard A.C., Churchack-Lichtin J.T. (2012). Preventive interventions for ADHD: A neurodevelopmental perspective. Neurotherapeutics, 9(3), 531–541. 10.1007/s13311-012-0123-z 22692794 PMC3441940

[44] Pesce C. (2009). An integrated approach to the effect of acute and chronic exercise on cognition: The linked role of individual and test constraints In Mc.Morris T., Tomporowski P.D., & Audiffren M. (Eds.), Exercise and cognitive function (pp. 213–226). Hoboken, NJ: Wiley.

[45] Chang Y.K., Liu S., Yu H.H., Lee Y.H. (2012). Effect of acute exercise on executive function in children with Attention–Deficit / Hyperactivity Disorder. Archives of Clinical Neuropsychology, 27, 225–237. 10.1093/arclin/acr094 22306962

[46] Den Heijer, A.E., Groen, Y., Tucha, L.I., Fuermaier, A.B.M., Koerts, J., Lange, K.W., et al., submitted.

[47] Hattori S., Naoi M., Nishino N. (1994). Striatal dopamine turnover during treadmill running in the rat: relation to speed of running. Brain Research Bulletin, 35(1), 41–49. 7953756 10.1016/0361-9230(94)90214-3

[48] Kim H., Heo H.I., Kim D.H., Ko I.G., Lee S.S., Kim S.E., et al (2011). Treadmill exercise and methylphenidate ameliorate symptoms of Attention–Deficit / Hyperactivity Disorder through enhancing dopamine synthesis and Brain-Derived Neurotrophic Factor expression in spontaneous hypertensive rats. Neuroscience Letters, 504(1), 35–39. 10.1016/j.neulet.2011.08.052 21907264

[49] Keijser, N., Piersma, D., Postema, F., Venema, B.J., Luiten, P.G.M., Riedel, G., et al. (2011). Improved cognitive performance as a result of whole body stimulation in mice and men. The 9th Dutch Endo-Neuro-Psycho (ENP) Meeting: 125.

[50] Lahr, M.M.H., Postema, F., Venema, B.J., Luiten, P.G.M., Riedel, G., van der Zee, E.A. (2009). Whole body stimulation functions as a cognitive enhancer in young and old mice. 8th Dutch Endo-Neuro-Psycho Meeting Abstract 170.

[51] Timmer M., Van der Zee E.A., Riedel G. (2006). Whole body vibration and behavior: Investigation of the role of various neurotransmitter systems. Federation of European Neuroscience Societies Abstract 3: 089.31.

[52] Van der Zee E.A., Riedel G., Rutgers E.H., De Vries C., Postema F., Venema B.J., et al (2010). Enhanced neuronal activity in selective brain regions of mice induced by whole body stimulation. Federation of European Neuroscience Societies Abstract 5: 024.49.

[53] Arias-Carrion O., Poeppel E. (2007). Dopamine, learning, and reward-seeking behavior. Acta Neurobiologiae Experimentalis, 67, 481–488. 18320725 10.55782/ane-2007-1664

[54] Arnsten A.F.T., Wang M.J., Paspalas C.D. (2012). Neuromodulation of thought: Flexibilities and vulnerabilities in prefrontal cortical network synapses. Neuron,76, 223–239. 10.1016/j.neuron.2012.08.038 23040817 PMC3488343

[55] Schultz W. (2007). Multiple dopamine functions at different time courses. Annual Review of Neuroscience, 30, 259–288. 17600522 10.1146/annurev.neuro.28.061604.135722

[56] Duan X., Wei S., Wang G., & Shi J. (2010). The relationship between executive functions and intelligence on 11- to 12-year-old children. Psychological Test And Assessment Modeling, 52(4), 419–431.

[57] Willcutt E.G., Doyle A.E., Nigg J.T., Faraone S.V., Pennington B.F. (2005). Validity of the executive function theory of Attention–Deficit / Hyperactivity–Disorder: a meta-analytic review. Biological Psychiatry, 57 (11), 1336–1346. 15950006 10.1016/j.biopsych.2005.02.006

[58] Dunn W. (1999). Sensory profile’s users manual. San Antonio, TX: Psychological Corporation.

