# Supplementary figures and images for: Acute Effects of Whole Body Vibration on Inhibition in Healthy Children
Source: PLoS One. 2015 Nov 2;10(11):e0140665. doi: 10.1371/journal.pone.0140665 (PMC4629895; doi:10.1371/journal.pone.0140665)

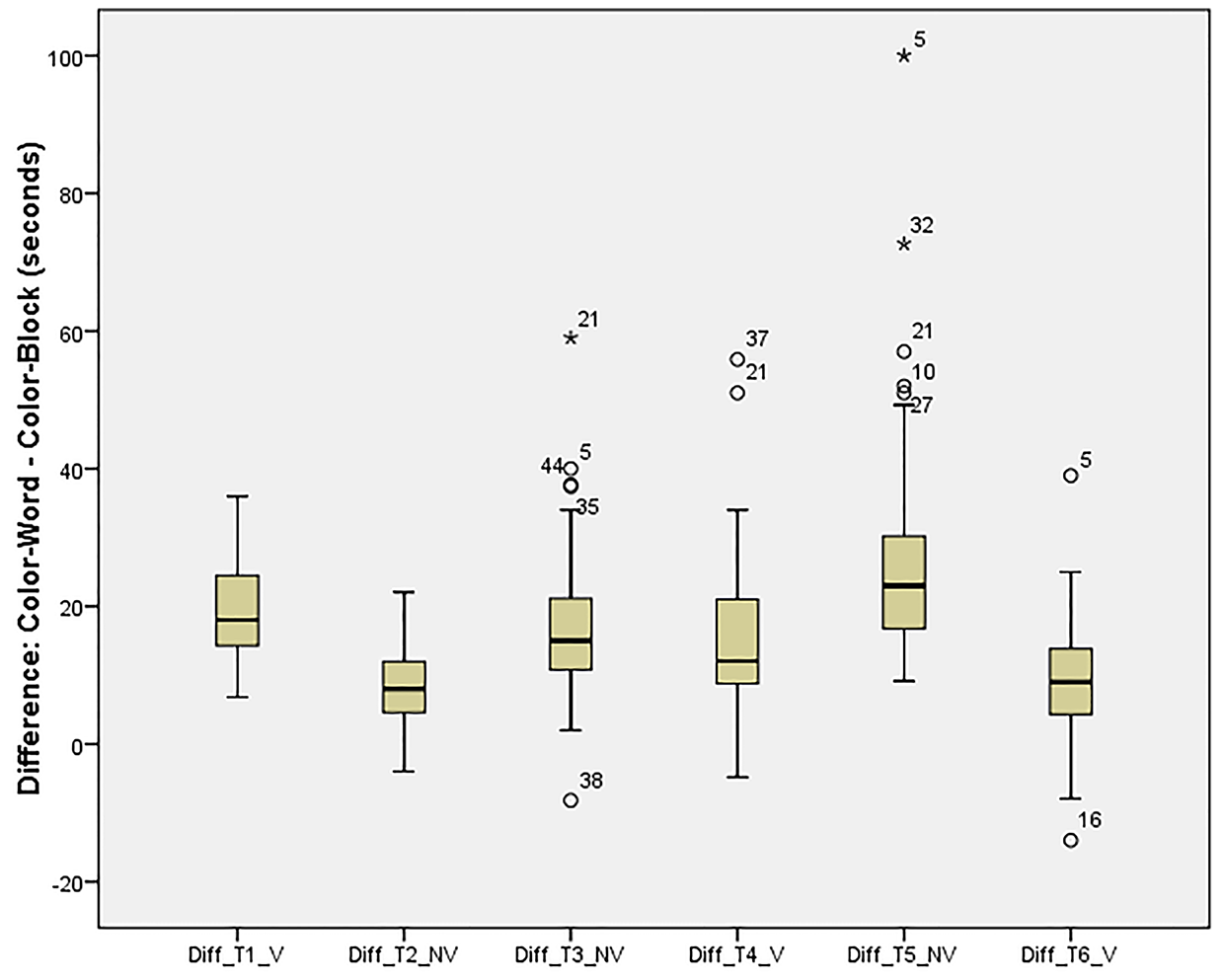

Supplement: S1 Fig — Distribution of the scores. (TIF) [file pone.0140665.s001.tif]
